# Nonperturbative approach to magnetic response of an isolated nanoring in a strongly anharmonic confinement

**DOI:** 10.1038/s41598-023-33544-x

**Published:** 2023-04-17

**Authors:** Y. J. Ding, Y. Xiao

**Affiliations:** 1 National Laboratory of Solid State Microstructure, School of Physics, Nanjing University, Nanjing, 210093 China; 2 College of Science, Nanjing University of Aeronautics and Astronautics, Nanjing, 210016 China

**Keywords:** Condensed-matter physics, Nanoscale materials, Theory and computation, Electronics, photonics and device physics, Computational science, Nanoscale devices

## Abstract

It is a huge challenge in both classical and quantum physics to solve analytically the equation of motion in a strongly anharmonic confinement. For an isolated nanoring, we propose a continuous and bounded potential model, which patches up the disadvantages of the usual square-well and parabolic potentials. A fully nonlinear and nonperturbative approach is developed to solve analytically the equation of motion, from which various frequency shifts and dynamic displacements are exactly derived by an order-by-order self-consistent method. A series of new energy levels and new energy states are found, indicating an alternative magnetic response mechanism. In nominally identical rings, especially, we observe a diamagnetic-paramagnetic transition in the period-halving Φ_0_/2-current with Φ_0_ the flux quantum and a large increase in the Φ_0_-current at least one order of magnitude, which explain well the experimental observations. This work opens a new way to solve the strong or weak nonlinear problems.

## Introduction

Nonlinear science has been seen as one of the most important frontiers for the fundamental understanding of nature. A particularly interesting example is the isolated nanoring in a strongly anharmonic confinement. A persistent current (PC) was expected to flow persistently even in non-superconducting metal rings without dissipation^1,2^. A series of experiments on metallic and semiconducting rings had indeed showed the evidence for the existence of PC^3–12^. In single Au ring, a paramagnetic PC was observed^4^ in period of flux quantum Φ_0_ = *h/e* with *h* Planck’s constant and *e* the electronic charge. In collective rings or arrays of rings^3,6,9^, however, the currents show a diamagnetic nature in period of Φ_0_- and/or Φ_0_/2. Some experiments measured the currents^3,9,10^ to be at least 1–2 orders of magnitude larger than prediction^13–15^, while other several experiments^5,8,11,12^ observed the currents in closer agreement with theory. Especially, Bluhm observed^11^ that both the direction and the magnitude of PC vary between nominally identical rings. Bleszynski-Jayich et al.^12^ experimentally confirmed diffusive non-interacting electrons in normal metal rings. Recently, the exact solution of a ring lattice model showed^16^ that a repulsive interaction changes the periodicity, the amplitude, and even the sign of PC at zero-temperature (*T* = 0). Therefore, the magnetic response of an isolated nanoring still remains a topic of controversy in condensed-matter physics^16–18^.

Theoretically, one-dimensional (1D) model ring predicted a priori random sign of PC^13–15,19,20^. Averaged over the electronic occupation, the Φ_0_/2-current shows a paramagnetic nature, in contradiction to the experiments^3,6^. Also, the period-halving Φ_0_/2-current was contributed to the spin degree of freedom^21^. Taken multi-channel effects into account, an analytical study on a finite width ring showed^22^ that a maximal paramagnetic and/or diamagnetic PC appears in primary Φ_0_- and/or Φ_0_/2-period with no requirement of ensemble average^19,20^ and electronic spin^21^, exhibiting a remarkable quantum size effect. This means that 1D model ring only represents an oversimplification of any real rings of finite width.

The importance of finite width was earlier recognized by Groshev et al.^23^ for describing the magnetic field dependence of PC. Subsequently, some two-dimensional (2D) model rings were widely used such as usual square well^24–26^ and parabolic potential^27–29^. For lack of a balance force, free electrons in a square well will be redistributed on the outer edge by the inertial centrifugal force, so that the potential is reconstructed and finally deviated from its initial one. For a parabolic potential, a local maximum exists at the ring center, which is very tiny for small radius of a nanoring. Only if Fermi energy exceeds this maximal value, the electrons would escape from the inner edge to the open ring center, meaning an insufficient capacity of electron filling. For their natural disadvantage, such 2D model rings are not so realistic for modeling a finite width ring with finite work-function.

In a real experiment, the number of electrons is typically 10^3^ in a semiconducting ring^30^, or much larger in a metallic ring^3^. To contain a large number of electrons, a strongly confined potential is needed for a sufficient capacity of electron filling. For its finite work-function, the ring-shaped potential for a nanoring changes abruptly from its bottom to vacuum environment, showing a strong nonlinearity. It is a huge challenge to solve analytically the equation of electronic motion in such a strongly nonlinear potential. This motivates us to develop a fully nonlinear and nonperturbative approach to explore the magnetic response of an isolated nanoring.

Considering the finite work-function, we propose a continuous and bounded potential for an isolated nanoring, which patches up the disadvantage of the usual square-well and parabolic potentials. To explore the intrinsic magnetic response of PC, for simplicity, in this work we would not consider the electronic spin and the electron–electron interactions, which had been reported previously^16,21^. A fully nonlinear and nonperturbative approach is developed to solve analytically the equation of electronic motion in the strongly anharmonic confinement, with no regular resonance divergence. The results show that the magnetic response of the isolated nanoring is inherently linked to the intrinsic sign of PC with barely any need of special size/disorder distributions over an array of mesoscopic rings. In nominally identical rings, especially, we observe a diamagnetic-paramagnetic transition in the Φ_0_/2-current and a large increase in the Φ_0_-current at least one order of magnitude, which explain well the experimental observations. The abrupt changes in the currents are mainly attributed to the newly found energy levels and energy states, revealing an alternative magnetic response mechanism.

## Model and method

### Strongly anharmonic confinement

For a 2D ring of radius ${r}_{c}$ and width *W* with width-diameter ratio $a=\frac{W}{2{r}_{c}}$, here we consider a continuous and bounded potential of strongly anharmonic confinement, $V={V}_{b}[\Gamma (x+a)-\Gamma \left(x-a\right)]$, where ${V}_{b}$ depicts the well depth and $\Gamma (x)=1/(1+{e}^{x/\upsigma })$ is a step-like function of *x*. Here $x=r/{r}_{c}-1$ is the relative radial coordinate of electron and σ is related to potential slope. For the later convenience of comparison, ${V}_{b}=\frac{{\hbar}^{2}{j}_{b}^{2}}{2m{r}_{c}^{2}}$ is expressed to have the same form as the angular kinetic energy of an electron in 1D ring with *m* the electron mass, where the dimensionless parameter *j*_*b*_ is equivalent to an angular quantum number. The potential has a reduced form

1 $$\mathrm{V}\left(x\right)=-\frac{{\mathrm{V}}_{\mathrm{b}}\mathrm{sh}\frac{a}{\upsigma }}{\mathrm{ch}\frac{a}{\upsigma }+\mathrm{ch}\frac{x}{\upsigma }}.$$

For an experimental ring^12^ of *r*_*c*_ = 418* nm* and *W* = 85* nm*, for example, $a\approx 0.1$, ${V}_{b}\approx 20meV$ and ${V}_{a}/{V}_{b}\approx -0.49331$ at *σ* = 0.04 and *j*_*b*_ = 300.

**Figure 1:**
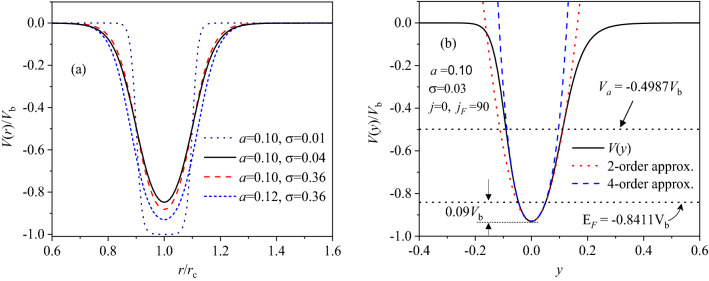
A continuous and bounded potential model for a nanoring of radius ${r}_{c}$ and width *W* under *j* = 0: (a) *V(r)* with various *a* and σ; (b) *V*(*y*) with *a* = 0.1 and σ = 0.03, as well as its 2- and 4-order polynomial approximants, where ${V}_{a}\approx -0.4987{V}_{b}$ and ${E}_{F}={V}_{c}+0.09{V}_{b}\approx -0.8411{V}_{b}$ at *j*_*F*_ = 90 and *j*_*b*_ = 300, as shown by two horizontal dotted lines.

As represented in Fig. 1, the potential has a static minimum of ${V}_{c}=-{V}_{b}\mathrm{sh}\frac{a}{\upsigma }/(\mathrm{ch}\frac{a}{\upsigma }+1)$ at $r={r}_{c}$ (*x* = 0), and is bounded from its bottom ${V}_{c}$ to zero far from $r={r}_{c}$. At ring edge, $V(a)=-\frac{1}{2}{V}_{b}th\frac{a}{\sigma }={V}_{a}$ at *x* =  ± *a*. Both ${V}_{c}$ and ${V}_{a}$ depend sensitively on both σ and *a*. For large σ, the potential approximates a parabolic confinement within the ring region (− *a* < *x* < *a*). For small σ, it gets close to a 2D square well, in which the electrons are almost free. For the electrons filling in all cases, Fermi energy ${E}_{F}={V}_{c}+\frac{{\hbar}^{2}{j}_{F}^{2}}{2m{r}_{c}^{2}}$ is referred to the potential bottom ${V}_{c}$, where *j*_*F*_ is a dimensionless parameter just as an angular quantum number in an ideal 1D ring.

Introducing $u=\frac{1}{r}$ and ${u}_{c}=\frac{1}{{r}_{c}}$, then $x =\frac{{u}_{c}}{u}-1=-\frac{y}{1+y}$ with $u={u}_{c}(1+y)$. The potential becomes a function of *y* only. For a larger σ (less slope), the potential may be well fitted by a parabolic approximation within the ring region. For a relatively small σ (larger slope), Fig. 1b shows *V(y)* for a nanoring of *a* = 0.1 and *σ* = 0.03, as well as its 2- and 4-order polynomial approximations. The asymmetry with respect of *y* = 0 can be clearly seen from Fig. 1b at higher energies. The potential is evidently deviated from the parabolic curve, where the outside confinement (*r* > *r*_*c*_*, y* < *0*) is substantially ‘harder’ than the inner one (*r* < *r*_*c*_*, y* > *0*). Within the ring region (*V* < *V*_*a*_), significantly, it is seen that the four-order polynomial approximation can well fit the model potential below a given ${E}_{F}$ at ${j}_{F}=90$ and ${j}_{b}=300$, despite a slight deviation at higher energies. This may simplify the later calculations and ensure the validity of the results. Especially, such a potential yields a nonlinear restoring force and thus the electrons will reside within the ring region, which is efficient and physically realistic for modeling the magnetic response of a finite width ring.

In the presence of magnetic flux Φ piercing through the ring center, the Hamiltonian of *non-interacting* electron in *spinless* case is given by $H=\frac{{p}_{r}^{2}}{2m}+{V}_{eff}$, where ${p}_{r}=-J\frac{du}{d\varphi }=-J\dot{u}$ is a radial momentum and ${V}_{eff}=\frac{{J}^{2}{u}^{2}}{2m}+V(y)$ is an effective potential. Here $J=j\hbar=(l+\phi )\hbar$ is the generalized angular momentum, *l* is the angular quantum number, and $\phi =\Phi /{\Phi }_{0}$ is a dimensionless flux. Using extreme value condition on *V*_*eff*_, the dynamic equilibrium point is relocated by

2 $${j}^{2}{u}_{0}^{3}=\frac{{j}_{b}^{2}{u}_{c}^{3}}{2\upsigma {w}_{0}^{2}}\mathrm{sh}\frac{a}{\upsigma }\mathrm{sh}\frac{\beta }{\upsigma },$$

at $x={u}_{c}/{u}_{0}-1=\beta$, where ${u}_{0}={u}_{c}/(1+\beta )$ and ${w}_{0}=ch\frac{a}{\sigma }+ch\frac{\beta }{\sigma }$. Then we get the extreme value ${V}_{0}=\frac{{J}^{2}{{\varvec{u}}}_{0}^{2}}{2m}-\frac{{V}_{b}}{{w}_{0}}sh\frac{a}{\sigma }$ at $u={u}_{0}$. By a simple iteration, it follows that $\beta \approx {\chi }^{2}{j}^{2}(1-3{\chi }^{2}{j}^{2})$, where $\chi =\frac{\sqrt{2}\sigma }{{j}_{b}}(ch\frac{a}{\sigma }+1)/{(sh\frac{a}{\sigma })}^{1/2}$ is a structural factor, determined only by the characteristic parameters of σ, *a* and *j*_*b*_. Obviously, the value of *χ* is very tiny at large ${j}_{b}$ and little *σ* (e.g., ${\chi \approx 5.47\times 10}^{-4}\ll 1$ at *σ* = 0.04, *a* = 0.1 and *j*_*b*_ = 300).

For a motion of small amplitude, the electron oscillates radially around *u* = *u*_0_, following in *u* = *u*_0_(1 + *y*). The Hamiltonian can be expressed in a Taylor series of *y*,

3 $$H={\mathrm{V}}_{0}+\frac{{\mathrm{J}}^{2}{u}_{0}^{2}{\dot{\mathrm{y}}}^{2}}{2m}+\frac{{\mathrm{J}}^{2}{u}_{0}^{2}}{m\beta }(\frac{1}{2}{\gamma }_{1}{\mathrm{y}}^{2}-\frac{1}{3}{\gamma }_{2}{\mathrm{y}}^{3}+\frac{1}{4}{\gamma }_{3}{\mathrm{y}}^{4}+\dots ),$$

with ${\gamma }_{1}\approx 1+4\beta$, ${\gamma }_{2}\approx 3+6\beta +\beta \frac{{w}_{0}-6}{2{\sigma }^{2}{w}_{0}}$, and ${\gamma }_{3}\approx 6+\frac{{w}_{0}-6}{6{\sigma }^{2}{w}_{0}}+\beta (10+\frac{3}{2{\sigma }^{2}}-\frac{15}{{\sigma }^{2}{w}_{0}})$. Upon the initial condition of *y*(0) = 0 and $\dot{y}$(0) = *η*, the electronic energy *E* is simply given by

4 $$E={\mathrm{V}}_{0}+\frac{{\mathrm{J}}^{2}{u}_{0}^{2}}{2m}{\upeta }^{2},$$

depending sensitively on the initial conditions of both *J* and *η*. The quantized solutions for *η* follow in a semi-classical description from the Bohr-Sommerfeld quantization rules^22,30^,

5 $$\oint {p}_{r}\mathrm{dr}=\oint \frac{\mathrm{J}{\dot{y}}^{2}\mathrm{d\varphi }}{{\left(1+y\right)}^{2}}=2\pi n\hbar,$$

with *n* the radial quantum number. Then we get from Eq. (4) the quantized energy levels *E*_*n,l*_($\phi$), each carrying a current *i*_*n,l*_ $=-\frac{1}{{\Phi }_{0}}\frac{\partial {E}_{n,l}}{\partial \phi }$. The total current *I*_*tot*_ is finally obtained by

6 $${I}_{tot}=\sum {i}_{n,l}=-\frac{1}{{\Phi }_{0}}\sum \frac{\partial {E}_{n,l}}{\partial\phi },$$

summing over *E*_*n,l*_ below Fermi energy *E*_*F*_ at *T* = 0. Fourier harmonics *A*_*k*_ of a current *I* are derived by ${A}_{k}={\int }_{-1/2}^{1/2}d\phi Isin(2\pi k\phi )$.

To examine the quantized solutions for $\eta$, the rest key task is to find the radial function *y* under its initial conditions. From Newton’s law, to the first order of *β*, we get the equation of motion,

7 $$\upbeta \ddot{y}+{\gamma }_{1}\mathrm{y}=f={\gamma }_{2}{\mathrm{y}}^{2}-{\gamma }_{3}{\mathrm{y}}^{3}+\cdots ,$$

where ***f*** acts as a nonlinear driving force. In a regular iterative method^31^, it is noticed that the even-order terms develop a constant average force (zero frequency), leading to a dynamic displacement, while the odd-order terms contain a base-frequency component, giving rise to a resonant divergence. That is, only if *f* ~ *sinφ, y* ~ *φcosφ* becomes divergent with *φ* → ∞. It is physically evident that the magnitude of the oscillation cannot increase of itself in a closed system with no external source of energy^32^. For the strong nonlinearity, it is a huge challenge to solve Eq. (7) analytically. One needs to develop a fully nonlinear and nonperturbative approach.

### Fully nonlinear and nonperturbative approach

To avoid the resonant divergence, we express the solution *y* as *y* = ∑*y*_*i*_ in a series of all-order trial solutions *y*_*i*_, meeting the initial conditions *y*_1_(0) = 0 and ${\dot{y}}_{1}$(0) = *η* while ${y}_{i}(0)={\dot{y}}_{i}$(0) = 0 at *i* > 1. Equation (7) is then rewritten into $\sum (\beta {\ddot{y}}_{i}+{\gamma }_{1}{y}_{i})={f}_{2}+{f}_{3}+\dots$, where *f* is classified by the power series into *f*_2_, *f*_3_, …, with ${f}_{2}={\gamma }_{2}{y}_{1}^{2}$ and ${f}_{3}=2{\gamma }_{2}{y}_{1}{y}_{2}-{\gamma }_{3}{y}_{1}^{3}$. Taking into account the nonlinear contributions of both the constant average force and the base-frequency component, we consider a generally trial solution of ${y}_{1}=\varepsilon +\frac{\eta }{\gamma }sin\gamma \varphi -\varepsilon cos\gamma \varphi =\varepsilon +Asin\theta$ with ${\mathrm{y}}_{1}$(0) = 0 and ${\dot{y}}_{1}$(0) = *η*. Here $A={(\frac{{\eta }^{2}}{{\gamma }^{2}}+{\varepsilon }^{2})}^\frac{1}{2}$, $\theta =\gamma \varphi +{\theta }_{0}$, and $tan{\theta }_{0}=-\varepsilon \gamma /\eta$. Two preset parameters of both *ε* and *γ* are introduced for the dynamic displacement and the frequency shift, which can be conveniently obtained by the order-by-order self-consistent approach.

At the linear approximation ($f\approx$ 0), it simply follows that $\upvarepsilon =0$, $\gamma ={\gamma }_{0}=\sqrt{\frac{{\gamma }_{1}}{\beta }}$ and $y\approx {y}_{1}=\frac{\eta }{{\gamma }_{0}}sin{\gamma }_{0}\varphi$. For small *β* (${\gamma }_{0}\gg 1$), the solution exhibits a *low-amplitude and high-frequency* oscillation. From this, the amplitude of high-order terms above the fifth in Eq. (3) can be roughly estimated by ${y}_{1}^{5}/\beta \sim {\beta }^{3/2}\sim {\chi }^{3}$, which may be very small and thus may be neglected. This means that we can obtain better accuracy only by considering the first few items. As a reference, the quantized solution for *η* is analytically obtained by $\frac{{}^{2}}{{\gamma }_{0}^{2}}=1-1/{(1+\frac{n}{{\gamma }_{0}j})}^{2}\approx \frac{2n}{{\gamma }_{0}j}$ at the linear approximation. The quantized energy levels are then given by ${E}_{n,l}\approx {V}_{0}+\frac{nJ{u}_{0}^{2}}{m}\sqrt{\frac{{\upgamma }_{1}}{\upbeta }}$, in approximately proportional to *n*, which is similar to that in a 2D parabolic potential^27–29^. For the higher-order approximation, the detailed derivations are given in Supplementary Information file.

Neglecting the higher-order terms, to the third-order approximation, the nonlinear driving force of $f\approx {f}_{2}+{f}_{3}$ involves not only the orbital-coupling-like effect $(\mathrm{e}.\mathrm{g}., 2{\gamma }_{2}{y}_{1}{y}_{2})$ but also the self-energy-like effect from the odd-order terms ($\mathrm{e}.\mathrm{g}., -{\gamma }_{3}{\mathrm{y}}_{1}^{3}$), both contributing to the average force and the base frequency. Defining $\mathrm{z}={\upgamma }^{2}/{\upgamma }_{0}^{2}$, both *ε* and *γ* (i.e., *z*) are exactly derived by

8 $$\varepsilon =\frac{1}{2}\frac{{\upeta }^{2}}{{\gamma }_{0}^{2}}\frac{{\gamma }_{2}}{{\gamma }_{1}},$$

9 $$(\mathrm{z}-1)(\mathrm{z}-\frac{1}{4})(\mathrm{z}-\frac{1}{9})=\frac{1}{3}\mu \frac{{\upeta }^{2}}{{\gamma }_{0}^{2}}\frac{{\gamma }_{2}^{2}}{{\gamma }_{1}^{2}}.$$

The dimensionless coefficients *κ* and *μ* are given by

10 $$\kappa=\frac{1}{z}(z-\frac{1}{4})(z-\frac{1}{9})/\left[{(z-\frac{1}{4})}^{2}(z-\frac{1}{9})+\frac{5}{6}{\Omega }_{0}\frac{{\upeta }^{2}}{{\gamma }_{0}^{2}}\right],$$

11 $$\upmu =1+2(\mathrm{z}-\frac{1}{4})\frac{{{\gamma }_{1}\gamma }_{3}}{{\gamma }_{2}^{2}}-\frac{9}{4}{}^{2}{\mathrm{z}}^{2}(\mathrm{z}-\frac{1}{4})(\mathrm{z}-\frac{1}{9}),$$

with ${\Omega }_{0}=\frac{1}{2}\frac{{\gamma }_{2}^{2}}{{\gamma }_{1}^{2}}+\frac{{\gamma }_{3}}{{\gamma }_{1}}(z-\frac{1}{4})$, both of which are only determined by variable *z*. Only if $\eta \ne 0$, it is necessitated that $z\ne 1$, $z\ne 1/4$, and $z\ne 1/9$, so that $\kappa \ne 0$, and $\mu \ne 0$. This means that the frequency shifts, the dynamic displacement, and even a series of new energy levels and new energy states can be expected due to the nonlinear resonance levels in such a confinement^32^, with no regular resonance divergence.

In essence, Eq. (9) is reducible to a ninth-order equation of *z*, which cannot be solved analytically. For a tiny amplitude of *η/γ*_0_, using an iterative approximation, we can solve Eq. (9) for *z* (i.e., γ) separately by sub-region at about *z* ~ 1, $z\sim \frac{1}{4}$, and $z\sim \frac{1}{9}$. Furthermore, *ε* can be obtained from Eqs.(8) and (10). The radial function of $y\approx {y}_{1}+{y}_{2}+{y}_{3}$ is specified by $y\approx {\uplambda }_{0}+{\uplambda }_{1}Asin\theta +{\uplambda }_{2}{A}^{2}cos2\theta +{\uplambda }_{3}{A}^{3}sin3\theta$, of which the parameters of both *A* and ${\uplambda }_{\mathrm{0,1},\mathrm{2,3}}$ depend on *ε* and *γ* (or *z*) and thus on *η/γ*_0_. Ignoring higher-order effects, *η* can be simply quantized by Eq. (5), and the quantized energy is then given by Eq. (4).

The total current can be further decomposed into three partial currents *I*_1,2,3_, originated from the levels contributions respectively at about *z* ~ 1, $z\sim \frac{1}{4}$, and $z\sim \frac{1}{9}$. The first current *I*_1_ just corresponds to PC in a parabolic potential, and the latter two currents *I*_2,3_ are induced by the newly found nonlinear resonance levels at about z = 1/4 and z = 1/9. While it is difficult to distinguish one from another, experimentally, three partial currents are measurable as a whole. Theoretically, the signs and the relative sizes of three partial currents will reveal the intrinsic magnetic response mechanism, which is different from that in the 1D ring, 2D square well, and parabolic potential.

In even higher approximations, nonlinear oscillations may also appear at other frequencies. As the degree of approximation increases, however, the oscillating strength decreases so rapidly that in practice only the first lower-order contribution can be observed^31^.

## Results and discussions

### Self-consistent iteration for *ε* and *γ*

From Eqs. (8)–(11), we can simply get all solutions for *ε* and *γ* by using self-consistent iteration. Defining $z-1=s$ at about z ~ 1, we get $\kappa\approx \frac{4}{3}$ and $\mu \approx \frac{3}{2}\frac{{{\gamma }_{1}\gamma }_{3}}{{\gamma }_{2}^{2}}-\frac{5}{3}$. Only a solution of *s* $\approx \frac{{\eta }^{2}}{{\gamma }_{0}^{2}}(\frac{3}{4}\frac{{\gamma }_{3}}{{\gamma }_{1}}-\frac{5}{6}\frac{{\gamma }_{2}^{2}}{{\gamma }_{1}^{2}})$ is derived by an iteration, and *ε* is then given by $\varepsilon \approx \frac{2}{3}\frac{{\eta }^{2}}{{\gamma }_{0}^{2}}\frac{{\gamma }_{2}}{{\gamma }_{1}}$. The quantized solution for *η* can be approximately obtained by $\frac{{\eta}^{2}}{{\gamma }_{0}^{2}}\approx \frac{2n}{{\gamma }_{0}j}(1-\frac{s}{2})$. Neglecting higher-order terms, this solution can be degenerated to that in the linear approximation.

Defining $z-\frac{1}{9}=s$ at about z ~ $\frac{1}{9}$, Eq. (9) is simplified into a *cubic* equation of *s*

12 $$\left(\mathrm{s}-\frac{3}{2}{\Omega }_{1}\frac{{\upeta }^{2}}{{\gamma }_{0}^{2}}\frac{{\gamma }_{2}}{{\gamma }_{1}}\right){\left(\mathrm{s}+12{\Omega }_{1}\frac{{\upeta }^{2}}{{\gamma }_{0}^{2}}\frac{{\gamma }_{2}}{{\gamma }_{1}}\right)}^{2}=\frac{3}{2}\frac{{9}^{3}}{{5}^{2}}{\mathrm{s}}^{3}\frac{{\upeta }^{2}}{{\gamma }_{0}^{2}}\frac{{\gamma }_{2}^{2}}{{\gamma }_{1}^{2}},$$

with ${\Omega }_{1}=\frac{9}{5}\frac{{\gamma }_{2}}{{\gamma }_{1}}-\frac{1}{2}\frac{{\gamma }_{3}}{{\gamma }_{2}}$. In a similar way, three solutions of $\mathrm{s}={s}_{1}\approx \frac{3}{2}{\Omega }_{1}\frac{{\upeta }^{2}}{{\gamma }_{0}^{2}}\frac{{\gamma }_{2}}{{\gamma }_{1}}$ and $\mathrm{s}={\mathrm{s}}_{2}\approx -12{\Omega }_{1}\frac{{\eta }^{2}}{{\gamma }_{0}^{2}}\frac{{\gamma }_{2}}{{\gamma }_{1}}(1\pm \frac{18\sqrt{3}}{5}\frac{\eta }{{\gamma }_{0}}\frac{{\gamma }_{2}}{{\gamma }_{1}})$ are newly obtained, having an additional contribution to PC, which are entirely absent in the usual 2D square well and parabolic potential. Then, *ε* is accordingly given by $\varepsilon ={\varepsilon }_{1}\approx -\frac{18}{5}\frac{{\eta }^{2}}{{\gamma }_{0}^{2}}\frac{{\gamma }_{2}}{{\gamma }_{1}}$ and $\varepsilon ={\varepsilon }_{2}\approx \mp 3\sqrt{3}\frac{\eta }{{\gamma }_{0}}(1\pm \frac{18\sqrt{3}}{5}\frac{\eta }{{\gamma }_{0}}\frac{{\gamma }_{2}}{{\gamma }_{1}})$. The quantized solutions for *η* can be approximately obtained by $\frac{{\eta}^{2}}{{\gamma }_{0}^{2}}\approx \frac{2n}{{\gamma }_{0}j}(1-\frac{9}{2}s)$ and $\frac{{\eta}^{2}}{{\gamma }_{0}^{2}}{(1\pm \frac{9\sqrt{3}}{2}\frac{\upeta }{{\gamma }_{0}}\frac{{\gamma }_{2}}{{\gamma }_{1}})}^{2}\approx \frac{2n}{{\gamma }_{0}j}(1-\frac{9}{2}s)$. To a *η*^3^-order approximation, obviously, the new energy levels appear a triple splitting at z ~ 1/9. Such a splitting is observable in high-precision spectral experiment, which may provide a check for validity of this model.

Defining $z-\frac{1}{4}=s$ at $z\sim \frac{1}{4}$, Eq. (9) is reduced to a *quintic* equation of *s*

13 $$(\mathrm{s}+\frac{16}{5}{\Omega }_{2})[{({\mathrm{s}}^{2}+3{\Omega }_{2})}^{2}-{\mathrm{s}}^{2}\frac{{\upeta }^{2}}{{\gamma }_{0}^{2}}\frac{{\gamma }_{2}^{2}}{{\gamma }_{1}^{2}}]=-\frac{16}{5}{\Omega }_{2}{\mathrm{s}}^{2}\frac{{\upeta }^{2}}{{\gamma }_{0}^{2}}\frac{{\gamma }_{2}^{2}}{{\gamma }_{1}^{2}},$$

with ${\Omega }_{2}=\frac{{\upeta }^{2}}{{\gamma }_{0}^{2}}(\frac{{\gamma }_{2}^{2}}{{\gamma }_{1}^{2}}+2\mathrm{s}\frac{{\gamma }_{3}}{{\gamma }_{1}})$. This means more new energy levels and new energy states appearing there, all of which are absent in the usual 2D square well and parabolic potential. From Eq. (13), we easily find its first solution of $s={s}_{1}\approx -\frac{16}{5}\frac{{\eta }^{2}}{{\gamma }_{0}^{2}}\frac{{\gamma }_{2}^{2}}{{\gamma }_{1}^{2}}$. In this case, we get $\varepsilon \approx -\frac{32}{15}\frac{{\eta }^{2}}{{\gamma }_{0}^{2}}\frac{{\gamma }_{2}}{{\gamma }_{1}}$, and the lowest-order response function is then derived by $y\approx \frac{5}{4}\frac{\eta }{{\gamma }_{0}}sin\theta +\frac{1}{4}\frac{\eta }{{\gamma }_{0}}\mathrm{sin}3\theta$, similar to a superposition state. The quantized solution for *η* can be obtained approximately by $\frac{{\eta}^{2}}{{\gamma }_{0}^{2}}\approx \frac{32}{17}\frac{n(1-2s)}{{\gamma }_{0}j}$, at variance with the linear approximation. The rest four solutions for *s* are approximately given by $\mathrm{s}={\mathrm{s}}_{2}\sim -\frac{\upeta }{{\gamma }_{0}}\frac{{\gamma }_{2}}{{\gamma }_{1}}[{\Omega }_{s}\pm {({\Omega }_{s}^{2}-3)}^\frac{1}{2}]$ with ${\Omega }_{s}=3\frac{\upeta }{{\gamma }_{0}}\frac{{\gamma }_{3}}{{\gamma }_{2}}\pm \frac{1}{2}$, where ${\Omega }_{s}^{2}\ge 3$ is required for a real solution. To find the new solutions possible for $\eta$, a variable $\Lambda$ is newly introduced by defining ${\Omega }_{s}=\sqrt{3} +{\Lambda }^{2}$, from which we get $\frac{\eta }{{\gamma }_{0}}=\frac{1}{3}(\sqrt{3} \mp \frac{1}{2}+{\Lambda }^{2})\frac{{\gamma }_{2}}{{\gamma }_{3}}$. This means some new energy levels existing at above a threshold value.

We now focus on the new energy levels yet to be set. Neglecting the higher-order terms, we get from Eq. (5) that $\frac{\eta }{{\gamma }_{0}}\approx {(\frac{2n}{{\gamma }_{0}j})}^\frac{1}{2}[1-{(\frac{2n}{{\gamma }_{0}j})}^\frac{1}{2}\frac{{\gamma }_{2}}{{\gamma }_{1}}(\sqrt{3} +{\Lambda }^{2}\pm {12}^\frac{1}{4}\Lambda )]$. In terms of two equivalent expressions of $\frac{\eta }{{\gamma }_{0}}$, the new variable $\Lambda$ is determined by

14 $$\Lambda =[{12}^\frac{1}{4}\frac{3n}{{\gamma }_{0}j}\frac{{\gamma }_{3}}{{\gamma }_{1}}\pm \sqrt{\Delta }]/(1+\frac{6n}{{\gamma }_{0}j}\frac{{\gamma }_{3}}{{\gamma }_{1}}).$$

For real solution of $\Lambda$, the discriminant $\Delta$ is required by

15 $$\Delta =18\sqrt{3}{(\frac{n}{{\gamma }_{0}j}\frac{{\gamma }_{3}}{{\gamma }_{1}})}^{2}+\left(1+\frac{6n}{{\gamma }_{0}j}\frac{{\gamma }_{3}}{{\gamma }_{1}}\right){\Omega }_{d}\ge 0,$$

with ${\Omega }_{d}={\Omega }_{\pm }=3{(\frac{2n}{{\gamma }_{0}j})}^\frac{1}{2}\frac{{\gamma }_{3}}{{\gamma }_{2}}-(\sqrt{3} \mp \frac{1}{2})-\frac{6\sqrt{3}n}{{\gamma }_{0}j}\frac{{\gamma }_{3}}{{\gamma }_{1}}$. The quantized solutions for $\eta$ are then obtained, and the quantized levels are correspondingly given by Eq. (4).

### Energy spectra and persistent current

**Figure 2:**
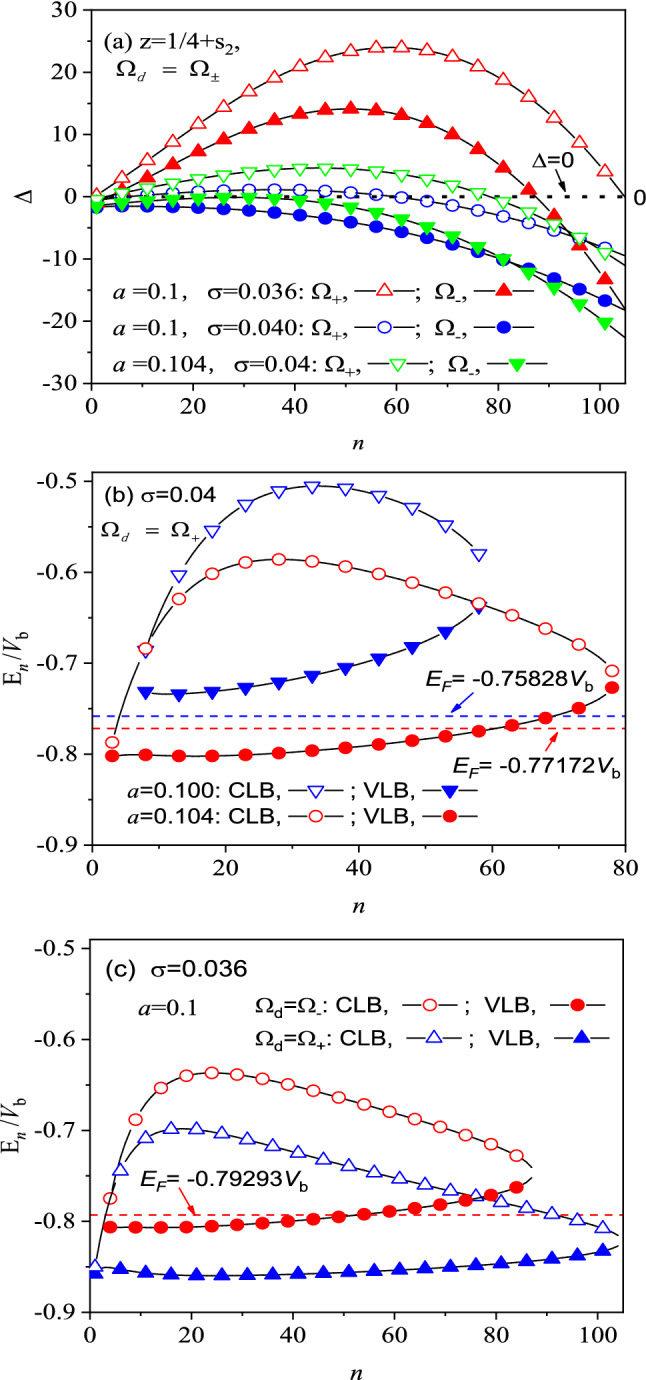
(**a**) The discriminant Δ and (**b**, **c**) the energy levels *E*_*n*_ as a function of *n* at $z=1/4+{\mathrm{s}}_{2}$ under *j* = 0 for various *a* and σ. Fermi energy is defined by ${E}_{F}={V}_{c}+0.09{V}_{b}$ at *j*_*F*_ = 90 and *j*_*b*_ = 300, as indicated by dashed lines in (**b**) and (**c**).

The existence of new energy states at $z=\frac{1}{4}+{s}_{2}$ depends directly on the sign of the discriminant Δ. The positive Δ-bands show those new energy levels existing stably, while the negative Δ-bands mean some unstable states. For the nanorings of various *a* and σ, as a typical example, we show in Fig. 2 the discriminant Δ and the stable energy levels *E*_*n*_ as a function of *n* for $z=\frac{1}{4}+{s}_{2}$ under *j* = 0. It is seen from Fig. 2a that some discrete positive Δ-bands are established within a finite interval of *n*, which may turn to be negative outside the *n*-interval. Obviously, only a positive Δ-band is observed at σ = 0.04, while two positive Δ-bands appear at σ = 0.036. The* n*-interval of σ = 0.04 becomes narrower at *a* = 0.1 than at *a* = 0.104, and even the total Δ-band may turn to be negative at less *a* and larger σ, no steady-state existing there. This shows that the ring’s geometry and characteristic play an important role on its energy levels and thus on its magnetic response.

In Fig. 2b and c, we further show the stable energy levels *E*_*n*_ for those positive Δ-bands. Fermi energy is given by ${E}_{F}-{V}_{c}=\frac{{\hbar}^{2}{j}_{F}^{2}}{2m{r}_{c}^{2}}=0.09{V}_{b}$ at *j*_*F*_ = 90 and *j*_*b*_ = 300, which is located within the ring region (*E*_*F*_ < *V*_*a*_). From Fig. 2b, we observe at σ = 0.04 that the positive Δ-band only for Ω_*d*_ = Ω_+_ corresponds to two discrete energy bands, one conduction-like band (CLB) and one valence-like band (VLB). The newly obtained energy states can be contributed to those nonlinear resonance levels^32^, modulated by the ring characteristic parameters (σ and *V*_*b*_). This also provides an intuitionistic physics image to understand the energy band theory of solid state physics. In the case of *a* = 0.104, most levels (*n* = 3–60) in VLB are lower than *E*_*F*_ (*E*_*F*_ =  − 0.77172*V*_*b*_), having major contribution to PC, while almost all levels in CLB are higher than *E*_*F*_, having hardly contribution to PC. In the case of *a* = 0.1, especially, it is found that a little reduction of width from *a* = 0.104 to *a* = 0.1 results in a large rise of the energy levels in both CLB and VLB, of which all levels are higher than *E*_*F*_ (*E*_*F*_ =  − 0.75828*V*_b_), having no contribution to PC. This can be easily understood by the fact that the energy levels may be approximated as those in a square well. As a result, the energy levels are largely enhanced by ${E}_{n}\sim 1/{W}^{2}\sim 1/{a}^{2}$, in inverse proportion to the square of the ring width, which exhibits a quantum size effect^22^.

In Fig. 2c, on the other hand, we explore the effect of the potential slope, where σ is changed from σ = 0.04 to σ = 0.036. Due to two positive Δ-bands at σ = 0.036, there appear two CLBs and two VLBs for Ω_*d*_ = Ω_±_. Most levels (*n* = 4–52) in the upper VLB and all levels in the lower VLB are lower than *E*_*F*_ (*E*_*F*_ =  − 0.79293*V*_*b*_), having additional contribution to PC. In fact, it is noticed that the coefficient ${\gamma }_{3}$ appearing in Eqs.(3,7,15) for anharmonic confinement depends strongly on the characteristic parameters σ and *a*, approximated by ${\gamma }_{3}\approx 6+\frac{{w}_{0}-6}{6{\sigma }^{2}{w}_{0}}$. This leads easily to the sign variation of the discriminant $\Delta$ in Eq. (15), and thus leads to the appearance of the new nonlinear resonance levels^32^ in favor of PC. The results indicate the PC’s sensitivity to both *a* and σ.

**Figure 3:**
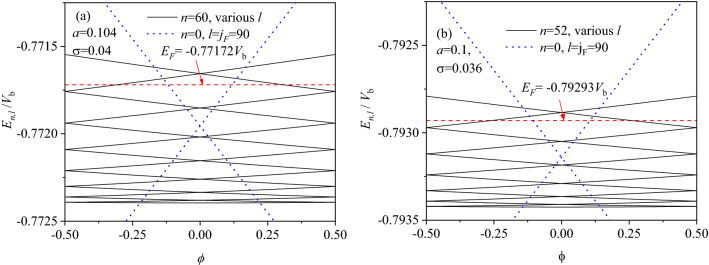
Energy levels *E*_*n,l*_ as a function of *ϕ* for various nanorings at $z=1/4+{s}_{2}$, where (**a**) *n* = 60 and (**b**) *n* = 52 correspond to the highest *E*_*n*_ in VLB below *E*_*F*_ in (**b**) and (**c**). For a comparison, Fermi band of *l* = *j*_*F*_ is present at *n* = 0 for the circle motion. *E*_*F*_ is defined just as in Fig. 2, indicated by red dashed lines.

In the presence of magnetic flux *ϕ*, to explore the magnetic response of PC, we show in Fig. 3 the energy spectrum *E*_*n,l*_(*ϕ*) of $z=\frac{1}{4}+{s}_{2}$ for various nanorings. For the main contribution of Fermi bands to the currents^22^, here we consider the highest levels of (*a*) *n* = 60 and (b) *n* = 52 in VLB below *E*_*F*_ in Fig. 2b and c. *E*_*F*_ is defined just as in Fig. 2. For comparison, Fermi band of *l* = *j*_*F*_ is present at *n* = 0 for the circle motion, similar to that in 1D ring. In the absence of radial motion (*n* = 0), it is seen that the energy level ${E}_{{j}_{F}}={V}_{0}<{\mathrm{E}}_{F}$ at *l* = *j*_*F*_ and *ϕ* = 0, as indicated by dotted lines. This is due to the quantum size effect that ${E}_{{j}_{F}}$ is depressed by ${u}_{0}={u}_{c}/(1+\beta )<{u}_{c}$, which is different from that in 1D ring of *u≡u*_*c*_. Interestingly, it is observed as a whole from Fig. 3 that the newly obtained energy levels show a dispersion similar to that in a parabolic potential. This may be due to the fact that in the presence of flux *ϕ*, an isolate level in an isolated nanoring behaves a similar dispersion. Thus the new energy states may have an equivalent or additional contribution to PC, which are entirely absent in the 1D ring, 2D square well and parabolic potential.

In the presence of radial motion (*n* ≠ 0), it is observed from Fig. 3 that each *l*-level is widen into an *l*-band with *ϕ* changing, forming the *n*th clustered bands for a given *n*. Within the Fermi band of *l* = *j*_*F*_ at *n* = 0, it is seen that the number of the various *l*-bands at a given *n* ≠ 0 is totally increased, while the *l-*band’s slopes are decreased. The current contribution of each *l*-band is accumulated to a substantial change, which cannot be ignored yet. Compared the σ = 0.04 and *a* = 0.104 ring in Fig. 3a with the σ = 0.036 and *a* = 0.1 ring in Fig. 3b, especially, it is observed that in the nominally identical nanorings, the band’s slopes and the band’s number below *E*_*F*_ are largely changed only by a little variation of both σ and *a*. It is just the band’s slopes and the band’s number below *E*_*F*_ that determine both the size and sign of PC, as defined by Eq. (6). This indicates an alternative magnetic response mechanism other than previous theory.

**Figure 4:**
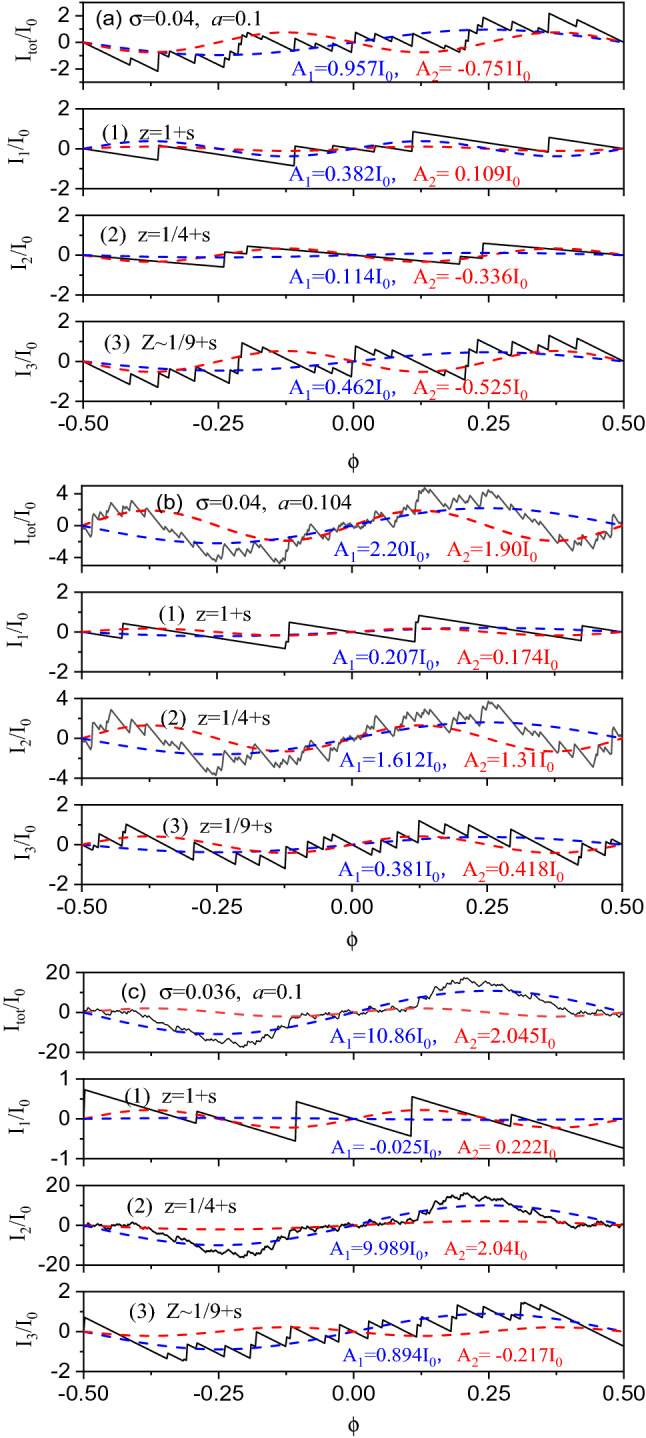
Total current *I*_*tot*_ and its partial currents *I*_1,2,3_ in units of *I*_0_ for the various nanoring of (**a**) *a* = 0.1 and σ = 0.04, (**b**) *a* = 0.104 and σ = 0.04, and (**c**) *a* = 0.1 and σ = 0.036. Below *I*_*tot*_-curves are three partial currents *I*_1,2,3_ for (1) *z* = 1 + *s*, (2) *z* = 1/4 + *s*, and (3) *z* = 1/9 + *s*. For the signs of PC, the first two Fourier harmonics *A*_1_ and *A*_2_ are present together.

Owing to uncertainty in experimental fabrication, in nature, there exists a fluctuation in both radius and width of collective rings^3,6^ or arrays of rings^9^. Also, the potential slope can be affected by substrate material, interface potential barrier and other experimental environment^3–12^. To explore the dependence of PC on σ and *a*, in Fig. 4 we show the total current *I*_*tot*_ and its partial currents *I*_1,2,3_ for various nanorings. Both *I*_*tot*_ and *I*_1,2,3_ are in units of ${I}_{0}={j}_{F}{\upomega }_{c}/{\Phi }_{0}$ with ${\omega }_{c}=\frac{\hbar}{m{r}_{c}^{2}}$. Here ${I}_{0}\sim 1.52nA$ can be estimated at ${j}_{F}=90$ and ${r}_{c}=418\mathrm{nm}$, and the obtained currents are basically within observation range^11,12^. For the direction of magnetic response, the first two Fourier harmonics *A*_1_ and *A*_2_ are present together to compare with the experimental data^11,12^.

In the case of the σ = 0.04 and *a* = 0.1 ring, three partial currents are never completely lost, despite having no current contribution of the positive Δ-band. It is seen from Fig. 4a that there appears a paramagnetic Φ_0_-current with *A*_1_ ~ 0.957*I*_0_ > 0, while the Φ_0_/2-current is diamagnetic with *A*_2_ ~  − 0.751*I*_0_ < 0. All three partial currents have positive contributions of *A*_1_ ~ 0.382*I*_0_, 0.114*I*_0_ and 0.462*I*_0_ to the Φ_0_-current of *A*_1_ ~ 0.957*I*_0_ in *I*_tot_, leading to a large paramagnetic Φ_0_-current. Strangely, the diamagnetic *Φ*_0_/2-current in *I*_tot_ originates mainly from the contributions of *A*_2_ ~  − 0.336*I*_0_ and − 0.525*I*_0_ in both *I*_2_ and *I*_3_, induced by the new energy levels, while the paramagnetic *Φ*_0_/2-current of *A*_2_ ~ 0.109*I*_0_ in *I*_1_ has been counterbalanced. This helps to explain the experimental observation on a diamagnetic *Φ*_0_/2-current^3,6^. The result is contributed to the newly found energy levels due to the nonlinear resonance, showing a new magnetic response mechanism.

In the case of the σ = 0.04 and *a* = 0.104 ring, both *Φ*_0_- and *Φ*_0_/2-currents are observed in Fig. 4b to be paramagnetic with *A*_1_ ~ 2.20*I*_0_ > 0 and *A*_2_ ~ 1.90*I*_0_ > 0. Comparing with Fig. 4a, a diamagnetic-paramagnetic transition appears in *Φ*_0_/2-current only by a little increase in width *a* from *a* = 0.1 to *a* = 0.104, showing a quantum size effect^22^. Also, it is seen that both *A*_1_ and *A*_2_ in *I*_tot_ are mainly governed by the contributions of both *A*_1_ ~ 1.612*I*_0_ and *A*_2_ ~ 1.31*I*_0_ in *I*_2_, while both *I*_1_ and *I*_3_ have a less contribution. This is due to the additional contribution in *I*_2_ from the positive Δ-bands.

In the case of the σ = 0.036 and *a* = 0.1 ring, a paramagnetic current appears in Fig. 4c with *A*_1_ ~ 10.86*I*_0_ > 0 and *A*_2_ ~ 2.045*I*_0_ > 0. For *A*_1_ being 5 times larger than *A*_2_, the total current shows up in primary Φ_0_ period, while its Φ_0_/2-current is counterintuitive to observe. In the same reason, both *A*_1_ and *A*_2_ in *I*_*tot*_ are governed by the contributions of both *A*_1_ ~ 9.989*I*_0_ and *A*_2_ ~ 2.04*I*_0_ from *I*_2_, while a weak diamagnetic current *A*_1_ ~  − 0.025I_0_ in *I*_1_ is completely counterbalanced and thus is unobservable. Under an identical width of *a* = 0.1, interestingly, it is found that only a little decrease in slope σ from σ = 0.04 to σ = 0.036 results in a large increase of *A*_1_ in *I*_*tot*_ from *A*_1_ ~ 0.957*I*_0_ to *A*_1_ ~ 10.86*I*_0_, the latter being at least one order of magnitude larger than the former. This is due to a huge contribution from two positive Δ-bands at σ = 0.036. The result can explain the difference in the PC’s magnitudes between the experiments^3,9,10^ and the predictions^13–15^. In nominally identical rings, especially, it is clearly seen that the direction and magnitude of PC depend sensitively on both ring width and potential slope, which explain well the experimental observation^11^. This further confirms the new magnetic response mechanism.

With σ decreasing (the slope increasing), actually, the model potential approximates a square well one. Even higher-order approximations may be needed to simulate such a potential, which also increases the difficulty of analysis. In this case, the magnetic response can be estimated at a relatively small σ. For a nanoring of *a* = 0.1 and *σ* = 0.032, both the paramagnetic Φ_0_- and Φ_0_/2-currents are approximately obtained to be *A*_1_ ~ 11.16*I*_0_ > 0 and *A*_2_ ~ 3.067*I*_0_ > 0. Compared to the case of the *a* = 0.1 and σ = 0.036 ring, the total current is not much affected by the slope, no significant alteration appearing there. From Eq. (15), furthermore, it is found that there exists a critical value of $\upsigma ={\upsigma }_{\mathrm{c}}$ at β = 0, so that ${\gamma }_{3}\approx 6+\frac{{w}_{0}-6}{6{\upsigma }^{2}{w}_{0}}>6( 6-\sqrt{3})$ at $\upsigma <{\upsigma }_{\mathrm{c}}$. For example, ${\upsigma }_{\mathrm{c}}\approx 0.03945$ is obtained at $a=0.1$. Then, the quadratic expression of ${\Omega }_{d}({n}^\frac{1}{2})>0$ and thus the discriminant $\Delta$>0, having a similar contribution to PC at $\upsigma <{\upsigma }_{\mathrm{c}}$. Such a result can also be related to the phase coherence length *L*_*ϕ*_ in a metallic ring of finite width, *L*_*ϕ*_ ~ 1* μm* achieved from theoretical fitting^23^ and *L*_*ϕ*_ > 2* μm* deduced from the experimental observation^3^, while the comparison between *L*_*ϕ*_ and PC may be not so straightforward. From this, it is expected that the current may become stable in a square-well potential at σ → 0.

## Conclusion

We have proposed a physically realistic potential model of a nanoring in a strongly anharmonic confinement, for which a fully nonlinear and nonperturbative approach is developed to solve analytically the equation of electronic motion, free from a regular resonance divergence. Both the frequency shift and the dynamic displacement are exactly derived by an order-by-order self-consistent method, leading to the findings of a series of new energy levels and new energy states. Also, the results of both CLB and VLB provide an intuitionistic physics image to understand the energy band theory of solid state physics. In nominally identical rings, especially, it is found that the direction and magnitude of PC depend sensitively on both ring width and potential slope, which explain well the experimental observations. The abrupt changes in the currents are mainly attributed to the newly found energy levels and energy states, revealing a new magnetic response mechanism. While the induced currents by the new energy levels cannot be measured independently, experimentally, the levels splitting at about z ~ 1/4 and z ~ 1/9 may be observable in high-precision spectral experiment. This may provide another check for the validity of the ring model and the new method. Considering the higher-order effects, further, some fine structures and even more new energy levels can be expected at other resonance frequencies^32^, of which the current contribution may be too weak to observe. In conclusion, this work opens another new way to solve the strong and/or weak nonlinear problems in both classical and quantum physics.

## Supplementary Information

Supplementary Information.

## Data Availability

All data generated or analysed during this study are included in this published article and its supplementary information files.
