# Supplementary material for: Nonperturbative approach to magnetic response of an isolated nanoring in a strongly anharmonic confinement
Source: Sci Rep. 2023 Apr 17;13:6235. doi: 10.1038/s41598-023-33544-x (PMC10110570; doi:10.1038/s41598-023-33544-x)
Supplement: Supplementary file 1 — Supplementary Information. [file 41598_2023_33544_MOESM1_ESM.pdf]

Supplementary Information

Nonperturbative approach to magnetic response of an isolated nanoring in a strongly anharmonic confinement

Y. J. Ding[1] and Y. Xiao[2]

[1] National Laboratory of Solid State Microstructure, School of Physics, Nanjing University, Nanjing 210093, China

[2] College of Science, Nanjing University of Aeronautics and Astronautics, Nanjing 210016, China

Exact solutions for parameters ε and γ

In the presence of the strongly anharmonic confinement, the parameters ε and γ for the dynamic displacement and the frequency shift can be conveniently obtained by an order-by-order self-consistent approach.

We express the solution y for Eq.(7) as $y=\sum y_i$ in a series of all-order trial solutions y_i, meeting the initial conditions $y_1(0)=0$ and $\dot{y}_1(0)=\eta$ while $y_i(0)=\dot{y}_i(0)=0$ at i>1. For the nonlinear contributions of both the constant average force and the base-frequency component, we consider a generally trial solution of $y_1=\varepsilon+\frac{\eta}{\gamma}sin\gamma\varphi-\varepsilon cos\gamma\varphi=\varepsilon+Asin\theta$ with $y_1(0)=0$ and $\dot{y}_1(0)=\eta$. Here $A=(\frac{\eta^2}{\gamma^2}+\varepsilon^2)^{\frac{1}{2}}$, $\theta=\gamma\varphi+\theta_0$, and $\tan\theta_0=-\varepsilon\gamma/\eta$. Eq.(7) is then rewritten into $\sum(\beta\ddot{y}_i+\gamma_1 y_i)=f_2+f_3+\cdots$, where f is classified by the power series into f_2, f_3, …, with $f_2=\gamma_2 y_1^2$ and $f_3=2\gamma_2 y_1 y_2-\gamma_3 y_1^3$.

For the linear approximation ($f\approx 0$), it simply follows that $\varepsilon=0$, $\gamma=\gamma_0=\sqrt{\frac{\gamma_1}{\beta}}$, and $y=\frac{\eta}{\gamma_0}sin\gamma_0\varphi$. For small β ($\gamma_0\gg 1$), the solution exhibits a *low-amplitude and high-frequency* oscillation.

For the second-order nonlinear approximation, $y_1=\varepsilon+Asin\theta$, $f\approx f_2=\gamma_2 y_1^2=\gamma_2(\varepsilon+Asin\theta)^2$, and $\beta\ddot{y}_1+\gamma_1 y_1=\gamma_1\varepsilon+(\gamma_1-\beta\gamma^2)\mathrm{A}\sin\theta$. Then we get that

$$\boldsymbol{\beta\ddot{y}_2+\gamma_1 y_2=f_2-(\beta\ddot{y}_1+\gamma_1 y_1)=\gamma_2\varepsilon^2-\gamma_1\varepsilon+\frac{1}{2}\gamma_2 A^2}$$
$$\boldsymbol{+(\gamma^2\beta-\gamma_1+2\varepsilon\gamma_2)A\sin\theta-\frac{1}{2}\gamma_2 A^2\cos 2\theta.} \qquad (\boldsymbol{S1})$$

Thus we can assume that $y_2=\mathrm{A}^2(d_0+d_1 sin\theta+d_2 cos2\theta)$ with $y_2(0)=\dot{y}_2(0)=0$. Substituting y_2 into Eq.(S1) and combining its initial conditions, we obtain that $d_0=-d_2(1+2sin^2\theta_0)$, $d_1=4d_2 sin\theta_0$ and $d_2=\frac{1}{8}\frac{\gamma_2}{\gamma_1}\frac{1}{z-1/4}$ with $z=\gamma^2/\gamma_0^2$. Both ε and γ are then determined by solving Eq.(S1) self-consistently. For the insufficient accuracy, however, we keep both ε and γ to be determined in the higher-order approximation.

Neglecting the higher-order terms, to the third-order approximation, Eq.(7) is then rewritten into

$$\boldsymbol{\beta \ddot{y}_3 + \gamma_1 y_3 = f_2 + f_3 - (\beta \ddot{y}_1 + \gamma_1 y_1) - (\beta \ddot{y}_2 + \gamma_1 y_2)}$$
$$\boldsymbol{= c_0 + c_1 \sin\theta + c_2 \cos 2\theta + c_3 \sin 3\theta,} \qquad (\boldsymbol{S2})$$

where the coefficients c_i (i=0, 1, 2, 3) are dependent on both ε and γ. In a similar way, we can suppose that $y_3 = A^3(a_0 + a_1 sin\theta + a_2 cos2\theta + a_3 sin3\theta)$ with $y_3(0) = \dot{y}_3(0) = 0$. Substituting y_3 into Eq.(S2) and combining its initial conditions, we get that $a_0 = -a_2(1 + 2sin^2\theta_0) - 8a_3 sin^3\theta_0$, $a_1 = 4a_2 sin\theta_0 - 3a_3(1 - 4sin^2\theta_0)$, $a_2 = -\frac{3\varepsilon\Omega_0}{8A}/(\mathrm{z} - \frac{1}{4})^2$, and $a_3 = -\frac{\Omega_0}{36}/(\mathrm{z} - \frac{1}{4})(\mathrm{z} - \frac{1}{9})$ with $\Omega_0 = \frac{1}{2}\frac{\gamma_2^2}{\gamma_1^2} + \frac{\gamma_3}{\gamma_1}(\mathrm{z} - \frac{1}{4})$.

For the dynamic displacement and the frequency shift, especially, the parameters ε and γ can be conveniently obtained by solving Eq.(S2) self-consistently, given by

$$\boldsymbol{\varepsilon[(z - \frac{1}{4})^2 - \frac{3}{2}\frac{\gamma_2}{\gamma_1}\varepsilon z(z - \frac{1}{4}) + \frac{3}{4}\frac{\eta^2}{\gamma_0^2}\frac{\gamma_2^2}{\gamma_1^2}(1 + 2(z - \frac{1}{4})\frac{\gamma_1\gamma_3}{\gamma_2^2})](z - \frac{1}{9})}$$
$$\boldsymbol{= \frac{1}{2}\frac{\eta^2}{\gamma_0^2}\frac{\gamma_2}{\gamma_1}(z - \frac{1}{4})(z - \frac{1}{9}) - \frac{5}{4}\varepsilon^3 z^2 \frac{\gamma_2^2}{\gamma_1^2}[1 + 2(z - \frac{1}{4})\frac{\gamma_1\gamma_3}{\gamma_2^2}],} \qquad (\boldsymbol{S3})$$

$$\boldsymbol{\varepsilon(z - 1)(z - \frac{1}{4})^2(z - \frac{1}{9}) - \frac{1}{3}\varepsilon\frac{\eta^2}{\gamma_0^2}\frac{\gamma_2^2}{\gamma_1^2}[1 + 2(z - \frac{1}{4})\frac{\gamma_1\gamma_3}{\gamma_2^2}](z - \frac{1}{4})}$$
$$\boldsymbol{= -\frac{3}{2}\varepsilon^2 z(z - \frac{1}{4})(z - \frac{1}{9})\frac{\gamma_2}{\gamma_1} + \frac{5}{4}\varepsilon^3 z^2 \frac{\gamma_2^2}{\gamma_1^2}[1 + 2(z - \frac{1}{4})\frac{\gamma_1\gamma_3}{\gamma_2^2}].} \quad (\boldsymbol{S4})$$

Both ε and γ, defined by Eqs.(8) and (9) in the text, are then derived exactly from Eqs.(S3) and (S4). The dimensionless coefficients κ and μ are given by Eqs.(10) and (11) in the text.

The radial function y is specified by $y \approx y_1 + y_2 + y_3 \approx \lambda_0 + \lambda_1 A sin\theta + \lambda_2 A^2 cos2\theta + \lambda_3 A^3 sin3\theta$ with $\lambda_0 = \varepsilon + A^2 d_0 + A^3 a_0$, $\lambda_1 = 1 + Ad_1 + A^2 a_1$, $\lambda_2 = d_2 + \mathrm{A}a_2$, and $\lambda_3 = a_3$. The parameters A and $\lambda_{0,1,2,3}$ depend on both ε and γ (or z) and thus on η/γ_0.

In even higher approximations, such non-linear oscillations may also appear at other frequencies. As the degree of approximation increases, however, the oscillating strength decreases so rapidly that in practice only the first lower-order contribution can be observed.
